# Association between marital status and in-hospital mortality in patients with acute coronary syndrome: a multivariable logistic regression analysis

**DOI:** 10.3389/fcvm.2026.1611679

**Published:** 2026-02-10

**Authors:** Lingling Zhang, Li Peng, Zhican Liu, Jianping Zeng, Xianghong Zhou, Ke Peng, Mingxin Wu, Mingyan Jiang

**Affiliations:** 1 Medical Department, Xiangtan Central Hospital, The Affiliated Hospital of Hunan University, Xiangtan, China; 2 Department of Oncology, Xiangtan Central Hospital, The Affiliated Hospital of Hunan University, Xiangtan, China; 3 Department of Pulmonary and Critical Care Medicine, Xiangtan Central Hospital, The Affiliated Hospital of Hunan University, Xiangtan, China; 4 Department of Cardiology, Xiangtan Central Hospital, The Affiliated Hospital of Hunan University, Xiangtan, China; 5 Department of Scientific Research, Xiangtan Central Hospital, The Affiliated Hospital of Hunan University, Xiangtan, China

**Keywords:** acute coronary syndrome, independent risk factor, in-hospital mortality, marital status, multivariable logistic regression, predictive model.

## Abstract

**Background:**

In patients with acute coronary syndrome (ACS), marital status may have a significant impact on the prognosis. However, it remains unclear whether marital status influences in-hospital mortality separately.

**Objective:**

This study aims to examine the relationship between marital status and in-hospital mortality in ACS patients and to develop a predictive scoring system using a multivariable logistic regression model to evaluate marital status as an independent risk factor for in-hospital mortality.

**Methods:**

We included 12,760 consecutive patients diagnosed with ACS during hospitalization. Patients were categorized into a death group or a survival group based on in-hospital outcomes, and further divided into married and non-married groups according to their marital status. Clinical data, including age, gender, ACS type, Killip classification, high-sensitivity troponin T (hsTnT), creatinine, and CKMB, were collected. We performed multivariable logistic regression analysis to assess the relationship between marital status and in-hospital mortality, and developed a predictive model. The model's performance was validated using ROC curve analysis and decision curve analysis (DCA).

**Results:**

Univariate analysis showed that being unmarried was significantly associated with higher in-hospital mortality (OR = 5.40, 95% CI: 3.26–8.95, *P* < 0.0001). Multivariable logistic regression confirmed this association (OR = 4.20, 95% CI: 1.93–9.10, *P* = 0.0003), indicating that marital status is an independent risk factor for in-hospital mortality. The ROC curve demonstrated a high predictive accuracy for the model, with an AUC of 0.972 (95% CI: 0.957–0.987). Decision curve analysis showed that the model including marital status provided the highest net benefit across most threshold probabilities. Based on these findings, we developed a nomogram scoring system incorporating marital status, age, ACS type, hsTnT, creatinine, CKMB, and Killip classification to predict in-hospital mortality risk.

**Conclusion:**

The marital status of ACS patients is an important independent predictor of in-hospital mortality. Unmarried patients have a significantly higher risk of in-hospital death.

## Introduction

Acute coronary syndrome (ACS) refers to a group of clinical conditions caused by insufficient blood flow to the coronary arteries, including unstable angina (UA), non-ST-elevation myocardial infarction (NSTEMI), and ST-elevation myocardial infarction (STEMI), all of which carry a high risk of in-hospital mortality (1–3). Although numerous studies have explored how various clinical and biomarker factors influence the prognosis of ACS patients (3–5), the impact of marital status as a potential psychosocial factor on in-hospital mortality has not been fully examined.

Marital status has been shown to significantly influence the prognosis of various diseases (6). Research indicates that married individuals generally fare better on several health measures compared to those who are single, divorced, or widowed, possibly due to the social support and emotional stability provided by marriage (7). However, most studies have focused on general cardiovascular disease populations, leaving the specific effects of different marital statuses (such as single, divorced, or widowed) on ACS patients less clear (8, 9).

This study aims to address this gap by systematically evaluating the influence of marital status on in-hospital mortality in ACS patients and exploring the potential mechanisms behind this relationship. We hypothesize that being unmarried (including single, divorced, or widowed) significantly increases the risk of in-hospital mortality among ACS patients. To test this hypothesis, we conducted a large-scale retrospective cohort study, using a multivariable logistic regression model to adjust for potential confounding factors and provide a more accurate risk assessment.

The novelty of this study lies in its first comprehensive analysis of how different marital statuses affect in-hospital mortality in ACS patients, using a large real-world registry and integrating marital status with detailed clinical characteristics to construct a multivariable risk assessment model. This research not only deepens our understanding of the role of marital status as a psychosocial factor in cardiovascular disease outcomes, but also offers new perspectives for identifying socially vulnerable patients and informing more personalized management strategies in clinical practice.

## Methods

### Study design and patient selection

**Figure 1:**
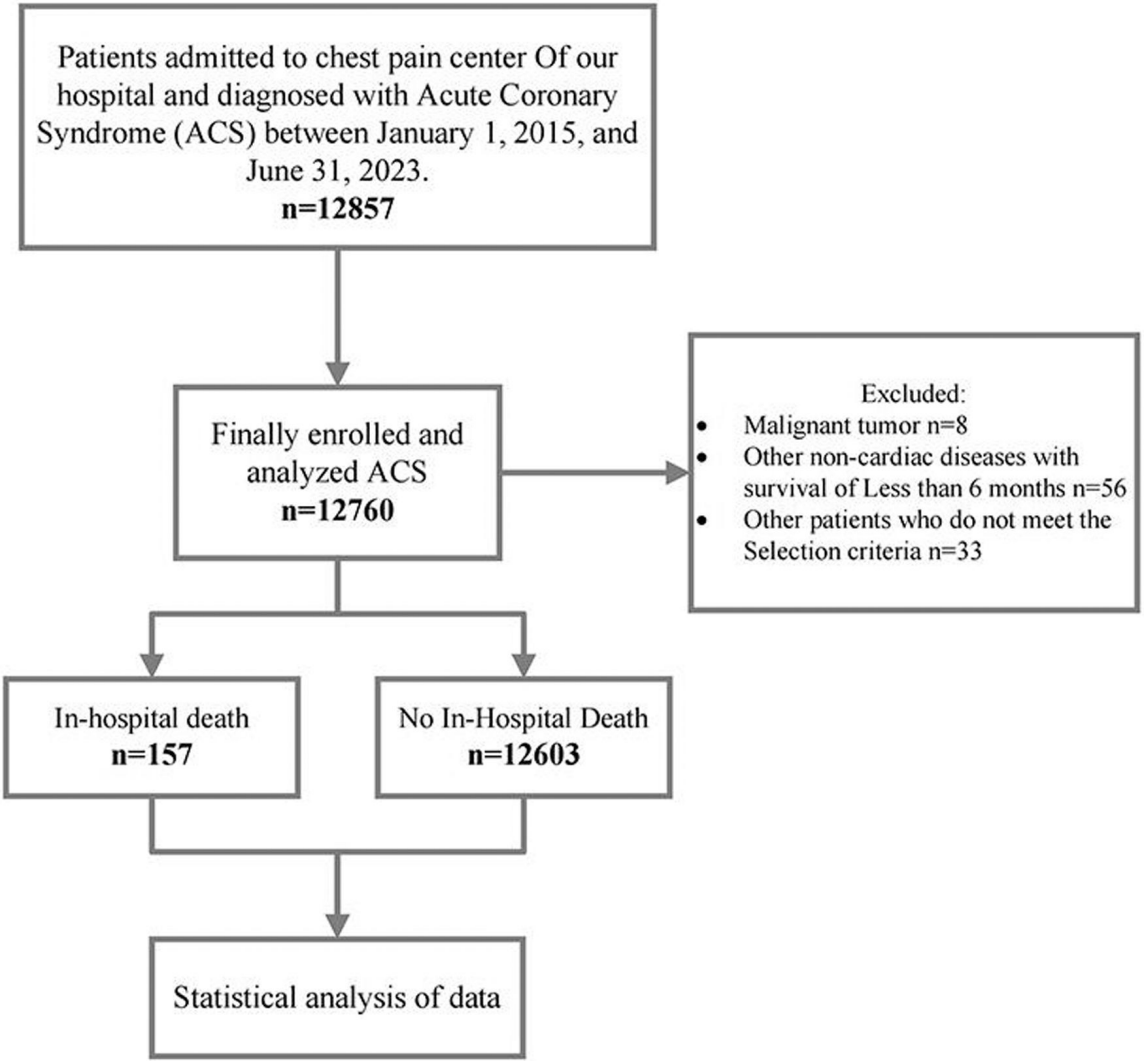
Flow diagram for participant screening, eligibility and analysis.

From January 2015 to June 2023, 12,857 patients with acute coronary syndrome (ACS) have been registered in a cardiac center's ACS registry (Figure 1). Inclusion Criteria: 1) Age ≥18 years; 2) Admitted to the hospital between January 2015 and June 2023 with a diagnosis of Acute Coronary Syndrome (ACS) according to the European Society of Cardiology (ESC) guidelines (1); 3) Availability of complete clinical and laboratory data. Exclusion Criteria: 1) Pregnant patients; 2) Concurrent diagnosis of malignant tumors; 3) Non-cardiac diseases with an expected survival of <6 months (e.g., end-stage liver disease, advanced pulmonary fibrosis); 4) Severe heart failure [New York Heart Association (NYHA) class III-IV]; 5) End-stage renal disease requiring dialysis; 6) Missing data or failure to meet other predefined inclusion criteria. ACS and other diagnoses (e.g., malignant tumors, COPD, stroke) were identified primarily from International Classification of Diseases, 10th Revision (ICD-10) codes recorded in the hospital information system by treating physicians and trained coding staff according to ESC guidelines and institutional coding rules.

### Data collection

Clinical data collected included age, gender, marital status, ACS type, Killip classification, high-sensitivity troponin T (hsTnT), creatinine, CKMB, and in-hospital mortality. At hospital admission, marital status was obtained using a standardized demographic registration form and was based on the patient's self-report (or information from family members or legal representatives when the patient was unable to provide it). This information is stored as a structured administrative variable in the electronic medical record. For the present analysis, marital status was categorized as married vs. unmarried (including single, divorced, and widowed). All data were extracted by trained researchers from the electronic medical records system and were double-checked for accuracy and completeness.

Data Access: The data were accessed for research purposes from July 1, 2023, to December 31, 2023. During this period, the authors did not have access to information that could identify individual participants, as all data were fully anonymized before analysis.

### Ethics and informed consent

The study protocol was approved by the Ethics Committee of Xiangtan Central Hospital (Xiangtan, China; Ethics Approval Number: 2023-02-001) and adhered to the principles outlined in the Declaration of Helsinki. As this study was retrospective and involved only the collection of clinical data without any interference in patient treatment plans, informed consent was waived.

### Statistical analysis

Statistical analyses were performed using SPSS 26.0 and R 4.1.2 software. Initially, all continuous variables were tested for normality. Variables that followed a normal distribution were expressed as mean ± standard deviation (SD) and compared between groups using independent sample t-tests. Non-normally distributed variables were presented as median (interquartile range) and compared using the Mann–Whitney U test. Categorical variables were reported as frequencies and percentages, with differences between groups assessed using the chi-square test or Fisher's exact test.

To evaluate the independent impact of marital status on in-hospital mortality, we first examined the association between each candidate variable and in-hospital mortality using univariate logistic regression. All clinically relevant potential predictors (demographic characteristics, cardiovascular risk factors and comorbidities, clinical presentation, laboratory indices, and marital status) were included in the univariate analyses. Variables with a *P*-value < 0.05 in the univariate models (16 out of 22 candidate variables) were then simultaneously entered into a multivariable logistic regression model. Adjusted odds ratios (ORs) with 95% confidence intervals (CIs) were reported. No formal correction for multiple testing was applied in the univariate screening step because these analyses were exploratory and intended only to select variables for inclusion in the multivariable model. The model's goodness-of-fit was assessed using the Hosmer-Lemeshow test.

The model's discriminatory ability was evaluated by constructing a receiver operating characteristic (ROC) curve and calculating the area under the curve (AUC). The model's stability was internally validated using the bootstrap method. Decision curve analysis (DCA) was conducted to assess the clinical utility of the model across different thresholds by calculating net benefit.

Based on the regression coefficients from the multivariable logistic regression model, a nomogram was developed using the “rms” package in R software. The nomogram translates the complex regression model into a simplified scoring system, making it easier for clinicians to use in practice. The predictive accuracy of the nomogram was evaluated with a calibration curve.

## Results

### Baseline characteristics

Table 1 compares the baseline characteristics between patients who experienced in-hospital mortality and those who survived. The mean age of patients who died in-hospital was 74.5 years (SD = 10.9), significantly higher than the 66.8 years (SD = 11.4) in those who survived (*P* < 0.001). The proportion of males was 61.1% in the in-hospital mortality group and 58.9% in the survival group, with no statistically significant difference (*P* = 0.572). Regarding marital status, 88.5% of patients who died in-hospital were married, and 8.9% were widowed, compared to 97.7% married and 0.9% widowed in the survival group—a difference that was statistically significant (*P* < 0.001).

**Table 1:** Baseline characteristics.

Variable	In-hospital death		*P*-value
	NO (*N* = 12,603)	YES (*N* = 157)	
Demographics			
Age, years	66.8 ± 11.4	74.5 ± 10.9	<0.001
Male, *N* (%)	7,425 (58.9%)	96 (61.1%)	0.572
Marital status			<0.001
Married	12,308 (97.7%)	139 (88.5%)	
Single	157 (1.2%)	2 (1.3%)	
Divorced	30 (0.2%)	2 (1.3%)	
Widowed	108 (0.9%)	14 (8.9%)	
Medical history, *N* (%)			
Atrial fibrillation	904 (7.2%)	28 (17.8%)	<0.001
Heart valve disease	2,257 (17.9%)	26 (16.6%)	0.661
Previous Myocardial infarction	880 (7.0%)	9 (5.7%)	0.541
Hypertension	8,602 (68.3%)	105 (66.9%)	0.713
Diabetes mellitus	3,763 (29.9%)	60 (38.2%)	0.023
COPD	2,423 (19.2%)	26 (16.6%)	0.399
Stroke	2,188 (17.4%)	49 (31.2%)	<0.001
Renal insufficiency	1,638 (13.0%)	74 (47.1%)	<0.001
Clinical conditions at admission			
LVEF, %	59.5 ± 11.0	49.6 ± 12.4	<0.001
NT-proBNP, pg/mL	1,712.4 ± 5,059.7	6,851.3 ± 11,084.0	<0.001
Troponin T, ng/mL	1.0 ± 2.2	4.0 ± 3.5	<0.001
Potassium,mg/L	4.1 ± 0.4	4.2 ± 0.5	0.002
Creatinine, mg/dL	89.1 ± 77.2	171.2 ± 157.4	<0.001
CKMB, U/L	37.9 ± 91.9	516.0 ± 212.0	<0.001
Hemoglobin, g/dL	130.4 ± 18.8	119.4 ± 22.3	<0.001
Low-Density Lipoprotein, mmol/L	2.6 ± 1.0	2.7 ± 1.0	0.081
Pulmonary infection, %	1,990 (15.8%)	75 (47.8%)	<0.001
ACS classification, %			<0.001
UA	8,728 (69.3%)	9 (5.7%)	
NSTEMI	2,300 (18.2%)	84 (53.5%)	
STEMI	1,575 (12.5%)	64 (40.8%)	
Killip classification, %			<0.001
0	8,728 (69.3%)	9 (5.7%)	
1	1,125 (8.9%)	4 (2.5%)	
2	1,371 (10.9%)	10 (6.4%)	
3	778 (6.2%)	19 (12.1%)	
4	601 (4.8%)	115 (73.2%)	

Categorical variables were presented as *n* (%). Values for continuous variables are given as means ± SD or medians with interquartile ranges.

COPD, Chronic Obstructive Pulmonary Disease; LVEF, left ventricular ejection fraction; NT-proBNP, N-terminal pro b-type natriuretic peptide; CKMB, Creatine Kinase-MB; ACS, Acute Coronary Syndrome; UA, Unstable Angina; NSTEMI, Non-ST Elevation Myocardial Infarction; STEMI, ST-Elevation Myocardial Infarction.

In terms of medical history, the rates of atrial fibrillation, diabetes, stroke, and renal insufficiency were significantly higher among patients who died in-hospital, at 17.8%, 38.2%, 31.2%, and 47.1%, respectively, compared to 7.2%, 29.9%, 17.4%, and 13.0% in those who survived (*P* < 0.001, *P* = 0.023, *P* < 0.001, *P* < 0.001). There were no significant differences between the two groups in other medical histories, such as valvular heart disease, prior myocardial infarction, hypertension, and chronic obstructive pulmonary disease. Clinically, the left ventricular ejection fraction (LVEF) was 49.6% (SD = 12.4) in patients who died in-hospital, significantly lower than the 59.5% (SD = 11.0) in those who survived (*P* < 0.001). Levels of NT-proBNP, troponin T, potassium, creatinine, CKMB, and hemoglobin were all significantly higher in patients who died in-hospital (*P* < 0.001). There was no significant difference in low-density lipoprotein levels between the two groups (*P* = 0.081). The incidence of pulmonary infection was 47.8% in patients who died in-hospital, significantly higher than the 15.8% in the survival group (*P* < 0.001). Regarding ACS classification, 53.5% of patients who died in-hospital had NSTEMI, 40.8% had STEMI, and 5.7% had UA; in contrast, in the survival group, 69.3% had UA, 18.2% had NSTEMI, and 12.5% had STEMI (*P* < 0.001). Killip classification revealed that 73.2% of patients who died in-hospital were Killip class 4, significantly higher than the 4.8% in the survival group (*P* < 0.001).

In supplementary analyses comparing baseline characteristics according to marital status (Supplementary Table S1), patients without a spouse were slightly younger than those with a spouse (65.1 ± 15.6 vs. 66.9 ± 11.3 years, *P* = 0.005), had a lower prevalence of hypertension (59.4% vs. 68.5%, *P* < 0.001), higher CKMB levels (71.9 ± 171.4 vs. 43.1 ± 105.9 U/L, *P* < 0.001), and were more likely to present with higher Killip class (Killip 4: 9.3% vs. 5.5%, overall *P* = 0.026). Other cardiovascular comorbidities and laboratory indices were generally comparable between married and unmarried patients.

### Impact of marital status on in-hospital mortality

Table 2 shows the results of the multivariable logistic regression analysis assessing the impact of marital status on in-hospital mortality among ACS patients. In Model A, which did not adjust for any variables, unmarried patients had a significantly higher risk of in-hospital mortality compared to married patients (OR = 5.40, 95% CI: 3.26–8.95, *P* < 0.0001). In Model B, which adjusted for gender and age, this association remained significant (OR = 5.57, 95% CI: 3.32–9.32, *P* < 0.0001). After further adjusting for ACS type, LVEF, NT-proBNP, troponin T, potassium, creatinine, CKMB, hemoglobin, low-density lipoprotein, Killip classification, atrial fibrillation, valvular heart disease, prior myocardial infarction, hypertension, diabetes, pulmonary infection, chronic obstructive pulmonary disease, stroke, and renal insufficiency in Model C, the association remained significant (OR = 4.12, 95% CI: 1.87–9.06, *P* = 0.0004).

**Table 2:** Impact of having a spouse on in-hospital mortality in ACS patients: multivariate logistic regression analysis.

Exposure	Model A		Model B		Model C	
	OR (95% CI)	*P*-value	OR (95% CI)	*P*-value	OR (95% CI)	*P*-value
Having Spouse						
Yes	Reference		Reference		Reference	
No	5.40 (3.26, 8.95)	**<0.0001**	5.57 (3.32, 9.32)	**<0.0001**	4.12 (1.87, 9.06)	**0.0004**

Bold represent significant values (*p* < 0.05).

Model A adjust for: None.

Model B adjust for: Sex; Age.

Model C adjust for: Sex; Age; ACS classification; LVEF; NT-proBNP; Troponin T; Potassium; Creatinine; CKMB; Hemoglobin; Low-Density Lipoprotein; Killip classification; Atrial fibrillation; Heart valve disease; Previous Myocardial infarction; Hypertension; Diabetes mellitus; Pulmonary infection; COPD; Stroke; Renal insufficiency.

OR: odds Ratio; CI: conﬁdence interval; other abbreviations can be found in Table 1.

The bold values indicate *P*-values less than 0.05.

**Figure 2:**
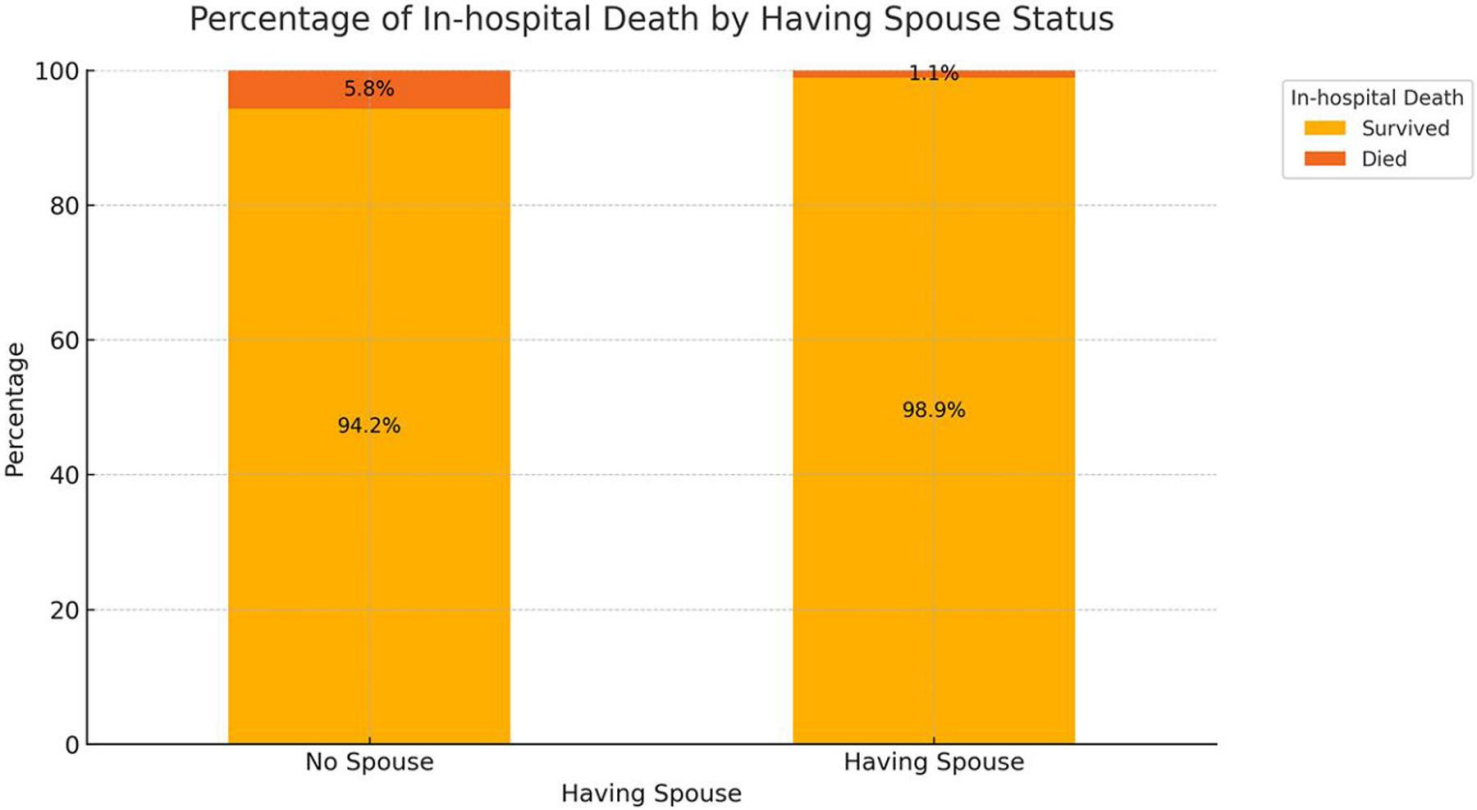
Impact of marital Status on in-hospital mortality.

Figure 2 shows the in-hospital mortality rates among patients with and without a spouse. The data indicate that in-hospital mortality was 5.8% among unmarried patients (including those who were single, divorced, or widowed), significantly higher than the 1.1% observed in married patients (*P* < 0.001). This suggests that marital status may be a key factor influencing in-hospital mortality.

### Univariate and multivariate logistic regression analysis

Table 3 presents the results of the univariate and multivariate logistic regression analyses examining factors associated with in-hospital mortality. In the univariate analysis, several factors were significantly linked to in-hospital mortality, including age (OR = 1.07, 95% CI: 1.06–1.09, *P* < 0.0001), troponin T (OR = 1.33, 95% CI: 1.28–1.38, *P* < 0.0001), CKMB (OR = 1.01, 95% CI: 1.01–1.01, *P* < 0.0001), NSTEMI (OR = 35.42, 95% CI: 17.78–70.54, *P* < 0.0001), STEMI (OR = 39.41, 95% CI: 19.57–79.34, *P* < 0.0001), unmarried status (OR = 5.40, 95% CI: 3.26–8.95, *P* < 0.0001), Killip class 2–4 (OR = 39.69, 95% CI: 22.46–70.12, *P* < 0.0001), creatinine (OR = 1.00, 95% CI: 1.00–1.00, *P* < 0.0001), stroke (OR = 2.16, 95% CI: 1.54–3.04, *P* < 0.0001), hemoglobin (OR = 0.97, 95% CI: 0.97–0.98, *P* < 0.0001), diabetes (OR = 1.45, 95% CI: 1.05–2.01, *P* = 0.0239), renal insufficiency (OR = 5.97, 95% CI: 4.34–8.20, *P* < 0.0001), pulmonary infection (OR = 4.88, 95% CI: 3.55–6.70, *P* < 0.0001), potassium (OR = 1.70, 95% CI: 1.22–2.37, *P* = 0.0016), NT-proBNP (OR = 1.00, 95% CI: 1.00–1.00, *P* < 0.0001), atrial fibrillation (OR = 2.81, 95% CI: 1.86–4.25, *P* < 0.0001), and LVEF (OR = 0.94, 95% CI: 0.93–0.95, *P* < 0.0001).

**Table 3:** Univariate and multivariate logistic regression analysis of factors affecting in-hospital mortality.

Exposure	Univariate Analysis		Multivariate Analysis	
	OR (95% CI)	*P*-value	OR (95% CI)	*P*-value
Age per year	1.07 (1.06, 1.09)	**<0**.**0001**	1.07 (1.04, 1.09)	**<0**.**0001**
Troponin T per ng/mL	1.33 (1.28, 1.38)	**<0**.**0001**	1.21 (1.14, 1.29)	**<0**.**0001**
CKMB per U/L	1.01 (1.01, 1.01)	**<** **0**.**0001**	1.01 (1.01, 1.01)	**<0**.**0001**
ACS classification				
UA	Reference		Reference	
NSTEMI	35.42 (17.78, 70.54)	**<** **0**.**0001**	41.74 (17.18, 101.41)	**<0**.**0001**
STEMI	39.41 (19.57, 79.34)	**<** **0**.**0001**	62.57 (25.58, 153.03)	**<0**.**0001**
Without Spouse vs. Having Spouse	5.40 (3.26, 8.95)	**<0**.**0001**	4.20 (1.93, 9.10)	**0**.**0003**
Killip classification 2–4 vs. 0–1	39.69 (22.46, 70.12)	**<0**.**0001**	11.81 (3.00, 46.48)	**0**.**0004**
Creatinine per mg/dL	1.00 (1.00, 1.00)	**<0**.**0001**	1.01 (1.01, 1.01)	**0**.**0060**
Stroke	2.16 (1.54, 3.04)	**<0**.**0001**	1.57 (0.97, 2.54)	0.0656
Hemoglobin per g/dL	0.97 (0.97, 0.98)	**<** **0**.**0001**	0.99 (0.98, 1.00)	0.1959
Diabetes mellitus	1.45 (1.05, 2.01)	**0**.**0239**	1.26 (0.79, 1.99)	0.3310
Renal insufficiency	5.97 (4.34, 8.20)	**<0**.**0001**	1.26 (0.74, 2.16)	0.3979
Pulmonary infection	4.88 (3.55, 6.70)	**<0**.**0001**	1.15 (0.72, 1.83)	0.5613
Potassium per mg/L	1.70 (1.22, 2.37)	**0**.**0016**	1.12 (0.74, 1.70)	0.5820
NT-proBNP per pg/mL	1.00 (1.00, 1.00)	**<0**.**0001**	1.00 (1.00, 1.00)	0.7058
Atrial fibrillation	2.81 (1.86, 4.25)	**<0**.**0001**	0.91 (0.50, 1.65)	0.7564
LVEF per 1%	0.94 (0.93, 0.95)	**<0**.**0001**	1.00 (0.98, 1.02)	0.9176
Low-Density Lipoprotein per mmol/L	1.15 (0.98, 1.34)	0.0807		
COPD	0.83 (0.55, 1.27)	0.4000		
Previous Myocardial infarction	0.81 (0.41, 1.59)	0.5417		
Male vs. Female	1.10 (0.79, 1.52)	0.5722		
Heart valve disease	0.91 (0.60, 1.39)	0.6616		
Hypertension	0.94 (0.67, 1.31)	0.7132		

OR, AbbreviatioSns can be found in Tables 1, 2.

All variables significantly associated with in-hospital mortality in the univariate analysis were included in the multivariate analysis. After adjusting for gender, age, ACS type, LVEF, NT-proBNP, troponin T, potassium, creatinine, CKMB, hemoglobin, low-density lipoprotein, Killip classification, atrial fibrillation, valvular heart disease, prior myocardial infarction, hypertension, diabetes, pulmonary infection, chronic obstructive pulmonary disease, stroke, and renal insufficiency, the following factors remained significantly associated with in-hospital mortality: age (OR = 1.07, 95% CI: 1.04–1.09, *P* < 0.0001), troponin T (OR = 1.21, 95% CI: 1.14–1.29, *P* < 0.0001), CKMB (OR = 1.01, 95% CI: 1.01–1.01, *P* < 0.0001), unmarried status (OR = 4.20, 95% CI: 1.93–9.10, *P* = 0.0003), Killip class 2–4 (OR = 11.81, 95% CI: 3.00–46.48, *P* = 0.0004), and creatinine (OR = 1.01, 95% CI: 1.01–1.01, *P* = 0.0060). Additionally, compared to patients with UA, those with NSTEMI (OR = 41.74, 95% CI: 17.18–101.41, *P* < 0.0001) and STEMI (OR = 62.57, 95% CI: 25.58–153.03, *P* < 0.0001) had significantly higher in-hospital mortality.

### Pairwise comparisons of marital status on in-hospital mortality

In crude comparisons across marital status categories (Supplementary Table S2), in-hospital mortality was 1.1% in married patients (139/12,447), 1.3% in single patients [2/159; relative risk (OR) 1.1, 95% CI 0.3–4.6; *P* = 0.867], 6.2% in divorced patients (2/32; OR 5.9, 95% CI 1.4–24.9; *P* = 0.016), and 11.5% in widowed patients (14/122; RR 11.5, 95% CI 6.4–20.5; *P* < 0.001) compared with married patients.

### Development of the predictive model

**Figure 3:**
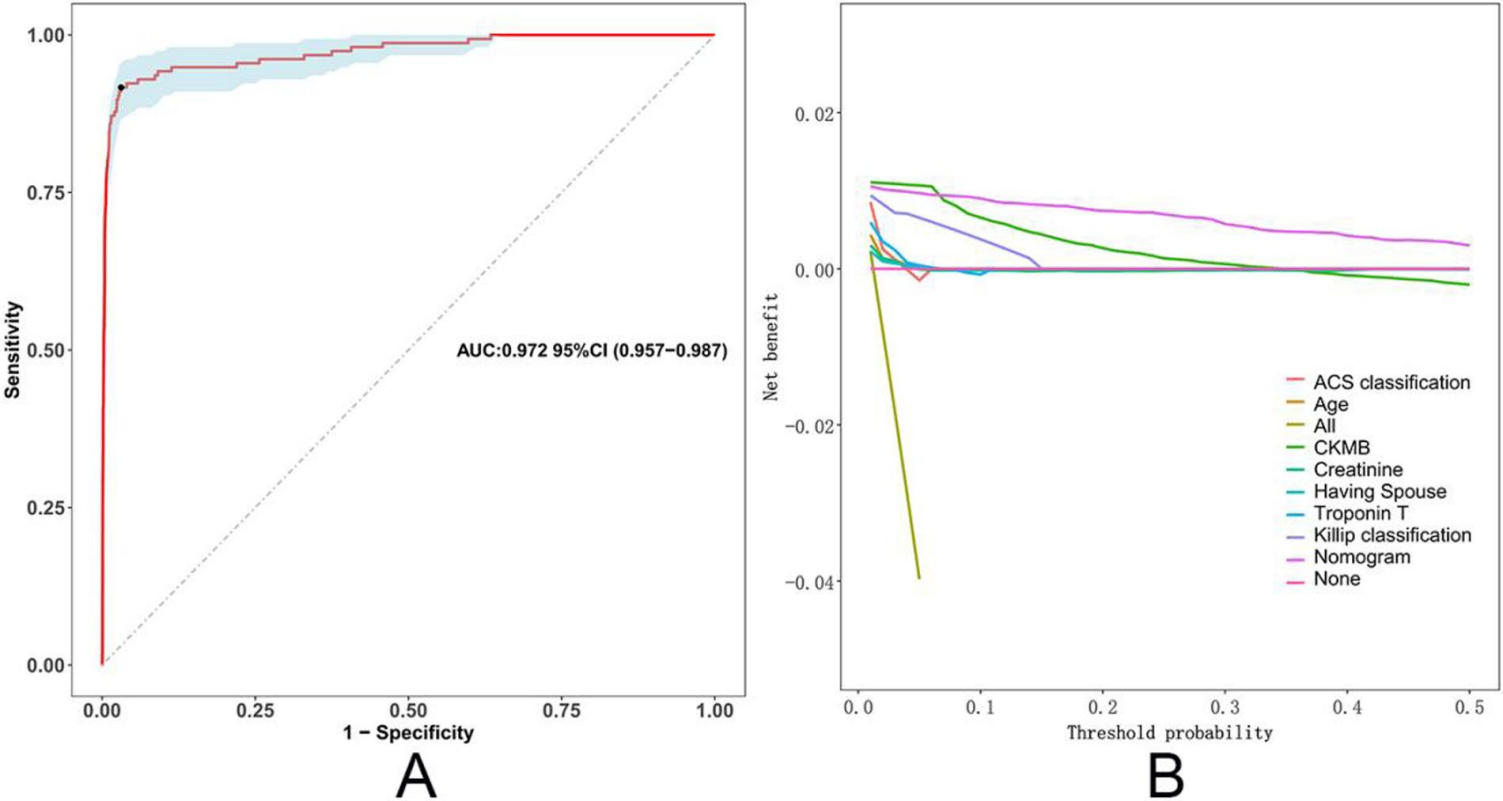
ROC curve and decision curve analysis of the in-hospital mortality predictive model. **(A)** The ROC curve illustrates the performance of the multivariable logistic regression model in predicting in-hospital mortality. **(B)** Decision curve analysis (DCA) evaluates the net benefit of the model across different threshold probabilities.

Figure 3A shows the ROC curve from the multivariable logistic regression model used to assess the accuracy of predicting in-hospital mortality. The model includes variables such as age, ACS classification (STEMI, NSTEMI, UA), high-sensitivity troponin T (hsTnT), creatinine, CKMB, Killip classification, and marital status. The ROC curve, which plots sensitivity against 1-specificity, demonstrates the model's performance across different thresholds. The area under the curve (AUC) is 0.972 (95% CI: 0.957–0.987), indicating a high level of discrimination, effectively distinguishing between high-risk and low-risk patients. Figure 3B presents the decision curve analysis (DCA), which evaluates the net benefit of different predictive models in clinical decision-making. The results show that the nomogram-based model offers the highest net benefit compared to individual variables.

**Figure 4:**
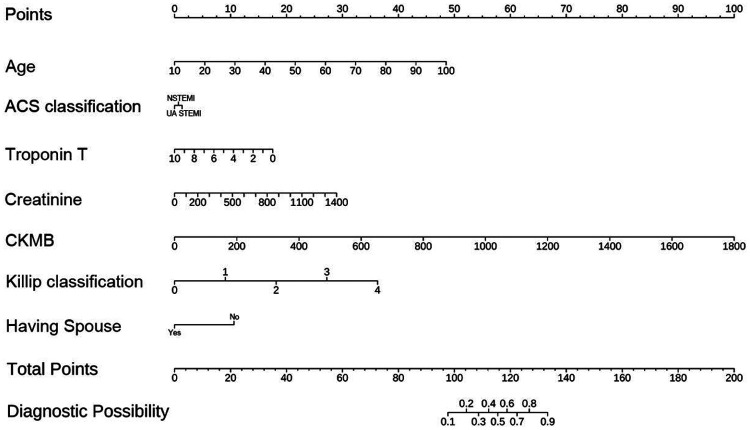
Nomogram for predicting in-hospital mortality.

Figure 4 displays the nomogram based on the multivariable logistic regression model for predicting in-hospital mortality in ACS patients. By identifying the corresponding score for each variable on the nomogram and summing these scores, a total score can be calculated for the patient. This total score can then be used to predict the patient's probability of in-hospital mortality. The nomogram simplifies complex statistical models, allowing clinicians to intuitively assess patient risk.

**Figure 5:**
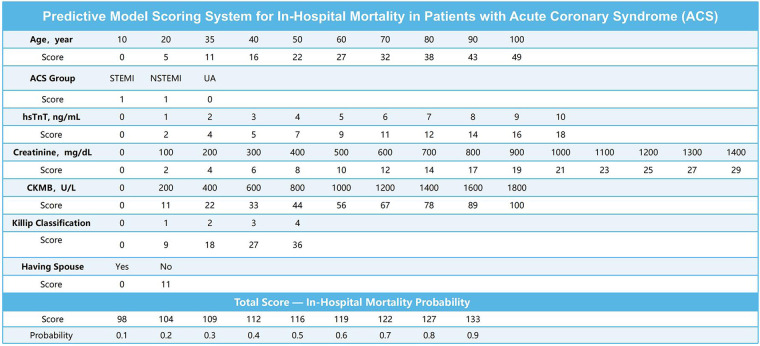
Scoring system for the in-hospital mortality predictive model.

Using the nomogram-derived scores, we developed a more user-friendly scoring tool (Figure 5) for predicting in-hospital mortality in ACS patients. The scoring system includes variables such as age, ACS classification (STEMI, NSTEMI, UA), high-sensitivity troponin T (hsTnT), creatinine, CKMB, Killip classification, and marital status. Each variable has an associated score on a scale provided in the figure. By locating the patient's specific value on each scale and summing the scores, a total score can be calculated. For example, a 70-year-old STEMI patient with an hsTnT level of 8 ng/mL, creatinine level of 1,000 mg/dL, CKMB level of 300 U/L, Killip classification of 3, and no spouse would receive the following scores:

- Age: 32 points
- ACS classification: 1 point
- hsTnT: 12 points
- Creatinine: 21 points
- CKMB: 12 points
- Killip classification: 27 points
- Marital status: 11 points

The total score is 116 points, corresponding to an in-hospital mortality probability of 60%. This scoring system provides clinicians with a straightforward method to assess the risk of in-hospital mortality in ACS patients and make informed treatment decisions.

To further illustrate the clinical usability of the model, we plotted the model-predicted probability of in-hospital mortality across the full range of total nomogram scores (Supplementary Figure S1). The curve shows that the estimated risk remains very low at lower scores and then increases steeply as the total score rises, indicating clear risk stratification, with patients with higher scores having a substantially greater predicted probability of in-hospital death.

## Discussion

This study explored the impact of marital status on in-hospital mortality among patients with acute coronary syndrome (ACS) and identified several key findings: being unmarried (including single, divorced, and widowed) significantly increased the risk of in-hospital mortality for ACS patients. The in-hospital mortality rate was 5.8% among unmarried patients, compared to 1.1% among married patients (*P* < 0.001). Among the unmarried group, widowed patients had the highest in-hospital mortality rate at 10.4%, followed by divorced patients at 5.1% and single patients at 4.9%. The predictive tool based on the multivariable logistic regression model demonstrated high accuracy, with an AUC of 0.972 (95% CI: 0.957–0.987) on the ROC curve. Decision curve analysis (DCA) further showed that the model incorporating all significant variables provided the highest net benefit across most threshold probabilities.

Given the low overall in-hospital mortality in this cohort (1.23%), the adjusted odds ratio of 4.20 for being without a spouse should therefore be interpreted primarily as a relative indicator of increased vulnerability rather than as a four-fold absolute increase in risk. Although the absolute difference in in-hospital mortality between unmarried and married patients (5.8% vs. 1.1%) appears numerically small, it is clinically meaningful in the context of contemporary low short-term mortality and highlights that the absence of a spouse identifies a subgroup at substantially higher risk within an otherwise low-risk population.

Our findings align with previous research, reinforcing the significant impact of marital status on cardiovascular outcomes (10, 11). Dupre et al. found that marital stability is closely linked to reduced mortality (9). Similarly, a systematic review and meta-analysis by Wong et al. emphasized the significant influence of marital status on the incidence and prognosis of cardiovascular disease (8). Takagi et al. also demonstrated that marriage might be associated with better postoperative outcomes in ACS patients (12). Prior studies have consistently shown that being married is associated with a lower risk of cardiovascular events and mortality, while being unmarried, particularly widowed, is linked to a higher risk of death (7). Our study not only supports these findings but also provides a more nuanced analysis of how different unmarried statuses (single, divorced, and widowed) differentially affect in-hospital mortality, with widowhood being associated with the highest risk. This increased risk may be related to the psychological stress and lack of social support following the loss of a spouse (6). Additionally, research has shown that married women or those living with a partner have a similar risk of ischemic heart disease but a significantly lower mortality rate from ischemic heart disease compared to unmarried women (13), likely due to the psychosocial benefits of marriage for women. However, some studies have offered different perspectives, suggesting that marriage might have potential adverse effects on health (14).

We now frame it more as: marital status provides a significant statistical and potentially clinically valuable signal for risk stratification, over and above conventional clinical and biomarker-based risk assessment. It likely serves as a composite marker representing a constellation of physiological, psychological, and social risks, rather than an effect in isolation.

There are several potential reasons why being unmarried significantly increases in-hospital mortality among ACS patients. First, social support plays a critical role in both psychological and physical health. Married patients typically benefit from better social support, which helps alleviate stress, improve psychological well-being, and promote healthy behaviors (15)—a factor that may be particularly pronounced in women (13). Research has shown that strong social support can lower stress hormone levels, enhance immune function, and improve cardiovascular health (16). Second, spousal support in disease management, such as encouraging adherence to medication, maintaining a healthy lifestyle, and attending regular medical appointments, can significantly improve patient outcomes (17). Moreover, marriage may provide economic support, enhancing access to healthcare resources and services, thereby improving treatment outcomes (18). Unmarried patients may lack these supports and resources, leading to poorer disease management and higher mortality risk. Widowed patients, in particular, may face greater psychological stress and loneliness, which can further exacerbate health problems (7).

While long-term mechanisms such as social support, health behavior monitoring, and economic advantages outside the hospital are crucial, our findings—that the association occurs during hospitalization—strongly suggest the existence of direct in-hospital pathways. Married patients may gain advantages during the acute in-hospital phase through several potential mechanisms:

First, regarding clinician-patient communication and advocacy, the presence of a spouse can aid in comprehending complex medical information, recalling treatment options, and more effectively communicating the patient's symptoms and preferences to the healthcare team. This role in “shared decision-making” and “patient advocacy” may ensure that clinical changes are promptly recognized and facilitate treatment adherence, thereby preventing delays or errors.

Second, in terms of providing practical physical and monitoring assistance, a spouse can offer crucial day-to-day care, such as helping with mobility, feeding, and medication intake. This not only improves patient comfort but may directly reduce the risk of in-hospital complications like falls, dehydration, or aspiration. Furthermore, as the closest observer, a spouse is often the first to detect subtle deteriorations in the patient's mental or physical condition and can alert medical staff promptly.

Finally, concerning the mitigation of acute psychological stress, ACS itself is an intensely stressful, traumatic event (19). The mere presence of a spouse serves as a powerful form of emotional support, helping to alleviate the patient's fear, anxiety, and sense of isolation. From a pathophysiological perspective, reducing acute psychological stress may help stabilize the autonomic nervous system and mitigate the catecholamine surge, thereby exerting a beneficial effect on heart rate, blood pressure, and myocardial oxygen demand (19, 20).

In conclusion, marital status provides not only a long-term health asset but also an immediately mobilizable “in-hospital protective resource” during acute illness. Future research should aim to directly observe and quantify these in-hospital interactive mechanisms.

The findings of this study should draw attention to the broader context of social determinants of cardiovascular health. Beyond marital status *per se*, factors such as social isolation, low socioeconomic status, and lack of social support have been consistently shown to exacerbate cardiovascular risk through multiple pathways (21–23). These pathways include the promotion of unhealthy behaviors (e.g., smoking, poor diet), amplification of chronic psychological stress (leading to autonomic dysfunction, HPA-axis activation, and increased inflammation), and barriers to accessing optimal healthcare due to financial or logistical obstacles. Thus, marital status can be viewed as a key and readily accessible marker of an individual's overall psychosocial support environment.

The impact of social factors on outcomes is particularly pronounced and frequently overlooked in female ACS patients. As highlighted by (24), women often present with different ACS symptoms, experience longer treatment delays, and have a risk profile more influenced by non-traditional factors like depression and low social support compared to men. Our findings gain particular significance in this context. Unmarried women, especially those widowed, may face a double burden of gender-specific health disparities and social isolation, positioning them as a highest-risk group meriting prioritized attention and targeted psychosocial interventions. Consequently, the association between marital status and outcomes observed in our study can be rationally framed within this broader context: marriage, as a key proxy for social support and economic stability, and its absence, may collectively contribute to adverse outcomes in ACS patients through the behavioral, physiological, and healthcare access pathways described above.

## Limitations of the study

This study has several limitations.

As a retrospective study, it is prone to selection bias and information bias. Although we made efforts to control for confounding factors, unmeasured potential confounders—including social and psychological variables that were not captured in the registry—may still have influenced the results. Future research should employ prospective cohort studies with richer data collection to minimize these biases. We did not formally test the linearity of continuous predictors on the logit scale or assess multicollinearity using variance inflation factors, and future model-building studies should incorporate these diagnostic procedures.

The classification of marital status in this study was relatively broad. For the main analysis, we dichotomized patients into “with a spouse” vs. “without a spouse” because the numbers of single, divorced and widowed patients with events were relatively small, which made separate modeling of these subgroups unstable. In addition, we were unable to collect detailed information on other aspects of social support and socioeconomic status, such as cohabitation with other family members or close companions, income, education level, or type of health insurance. Therefore, marital status should be regarded as a crude proxy for social support, and residual confounding by unmeasured social and socioeconomic factors cannot be excluded.

The data for this study were collected from a single center, and the external validity of the results needs to be confirmed in different populations and regions. Multi-center studies will be essential to validate the generalizability of these findings. Additionally, while our model showed high apparent predictive accuracy, further adjustments, internal validation with optimism-corrected performance measures, and external validation in independent cohorts are required before it can be used routinely in clinical practice.

The primary limitation of using ICD-10 codes to define our cohort is their inability to precisely capture disease severity and specific phenotypes. While this might affect the precision of the cohort definition, we believe the core findings remain robust, supported by their alignment with objective laboratory and clinical data.

This study is susceptible to temporal bias due to its extended duration (2015–2023). During this period, significant advancements were made in the management of ACS, particularly in revascularization techniques and evidence-based pharmacotherapy. Because of the constraints of our database, we were unable to collect and include patient-level data on specific treatments (e.g., precise percutaneous coronary intervention strategies, use of new-generation drug-eluting stents, or details of guideline-directed medical therapy), and we therefore cannot fully rule out the possibility of residual confounding related to changes in treatment over time.

### Future research directions

The scope of the present study was restricted to outcomes during the hospitalization period. Future work will include long-term follow-up, which is crucial for distinguishing if social support functions solely in the acute setting or provides sustained protection over time. This deeper understanding would substantially strengthen the interpretation and impact of our research.

Future research should further explore the mechanisms linking marital status with ACS prognosis, particularly the impact of different types of marital arrangements on health. Prospective cohort studies and randomized controlled trials will be crucial in validating our findings and guiding the development of interventions. Moreover, interdisciplinary research that integrates psychology and sociology will provide a more comprehensive understanding of the impact of psychosocial factors on cardiovascular outcomes. Research should also investigate whether interventions, such as social support programs and psychological counseling, can reduce in-hospital mortality among unmarried ACS patients. These studies will provide valuable insights for clinical practice, helping to develop more effective intervention strategies and improve the overall prognosis of ACS patients.

## Conclusion

This study demonstrates that marital status is an important independent predictor of in-hospital mortality among ACS patients. Unmarried patients have a significantly higher risk of in-hospital mortality compared to married patients. Based on this, we developed a multivariable predictive model and a nomogram scoring system that includes marital status, providing clinicians with a simple and effective tool for assessing in-hospital mortality risk in ACS patients and developing personalized treatment strategies. Further research is needed to validate and expand on our findings.

## Data Availability

The raw data supporting the conclusions of this article will be made available by the authors, without undue reservation.

## Supplementary material

Supplementary Table S1 Baseline Demographic and Clinical Characteristics of ACS Patients According to Marital Status (With vs Without a Spouse).

Supplementary Table S2 Crude in-hospital mortality and relative risk of death according to marital status.

Supplementary Table S3 Coefficients and multicollinearity diagnostics (variance inflation factors, VIFs) for predictors in the final multivariable model.

Supplementary Figure S1 Predicted probability of in-hospital mortality according to total nomogram score.
