# Supplementary material for: Association between marital status and in-hospital mortality in patients with acute coronary syndrome: a multivariable logistic regression analysis
Source: Front Cardiovasc Med. 2026 Feb 10;13:1611679. doi: 10.3389/fcvm.2026.1611679 (PMC12929463; doi:10.3389/fcvm.2026.1611679)

| **Supplementary Table 1. Baseline Demographic and Clinical Characteristics of ACS Patients According to Marital Status (With vs Without a Spouse)** | | | |
| --- | --- | --- | --- |
|  | Having Spouse | | *P-value* |
|  | YES（N=12447) | NO(N=313) |  |
| **Demographics** |  |  |  |
| Age, years | 66.9 ± 11.3 | 65.1 ± 15.6 | 0.005 |
| Male, N (%) | 7347 (59.0%) | 174 (55.6%) | 0.222 |
| **Medical history, N (%)** |  |  |  |
| Atrial fibrillation | 911 (7.3%) | 21 (6.7%) | 0.682 |
| Heart valve disease | 2239 (18.0%) | 44 (14.1%) | 0.073 |
| Previous Myocardial infarction | 873 (7.0%) | 16 (5.1%) | 0.192 |
| Hypertension | 8521 (68.5%) | 186 (59.4%) | <0.001 |
| Diabetes mellitus | 3735 (30.0%) | 88 (28.1%) | 0.47 |
| COPD | 2398 (19.3%) | 51 (16.3%) | 0.187 |
| Stroke | 2183 (17.5%) | 54 (17.3%) | 0.895 |
| Renal insufficiency | 1669 (13.4%) | 43 (13.7%) | 0.866 |
| **Clinical conditions at admission** |  |  |  |
| LVEF, % | 59.4 ± 11.1 | 58.7 ± 11.4 | 0.261 |
| NT-proBNP, pg/ml | 1776.1 ± 5199.9 | 1755.7 ± 5474.8 | 0.945 |
| Troponin T, ng/mL | 1.0 ± 2.2 | 1.2 ± 2.5 | 0.125 |
| Potassium,mg/L | 4.1 ± 0.4 | 4.1 ± 0.4 | 0.751 |
| Creatinine, mg/dL | 89.9 ± 78.4 | 95.6 ± 106.2 | 0.207 |
| CKMB, U/L | 43.1 ± 105.9 | 71.9 ± 171.4 | <0.001 |
| Hemoglobin, g/dL | 130.3 ± 18.8 | 129.6 ± 21.7 | 0.49 |
| Low-Density Lipoprotein, mmol/L | 2.6 ± 1.0 | 2.6 ± 0.9 | 0.931 |
| Pulmonary infection, % | 2008 (16.1%) | 57 (18.2%) | 0.324 |
| ACS classification, % |  |  | 0.507 |
| UA | 8532 (68.5%) | 205 (65.5%) |  |
| NSTEMI | 2319 (18.6%) | 65 (20.8%) |  |
| STEMI | 1596 (12.8%) | 43 (13.7%) |  |
| Killip classification, % |  |  | 0.026 |
| 0 | 8532 (68.5%) | 205 (65.5%) |  |
| 1 | 1100 (8.8%) | 29 (9.3%) |  |
| 2 | 1355 (10.9%) | 26 (8.3%) |  |
| 3 | 773 (6.2%) | 24 (7.7%) |  |
| 4 | 687 (5.5%) | 29 (9.3%) |  |
| Abbreviations: OR: Abbreviations can be found in Table 1 | | | |

| **Supplementary Table 2. Crude in-hospital mortality and relative risk of death according to marital status** | | | |
| --- | --- | --- | --- |
| Marital status | death/Total（n%） | OR (95% Confidence Interval) | *P-value* |
| Married | 139/12447 (1.1%) | Reference |  |
| Single | 2 /159(1.3%) | 1.1 (0.3, 4.6) | 0.867 |
| Divorced | 2 /32(6.2%) | 5.9 (1.4, 24.9) | 0.016 |
| Widowed | 14/122 (11.5%) | 11.5 (6.4, 20.5) | <0.001 |

| **Supplementary Table 3.Coefficients and multicollinearity diagnostics (variance inflation factors, VIFs) for predictors in the final multivariable model.** | | |
| --- | --- | --- |
|  | Coefficients | VIF |
| Age | 0.0665 | 1.0449 |
| ACS classification | 0.0825 | 1.2972 |
| Troponin T | -0.2162 | 1.6951 |
| Creatinine | 0.0025 | 1.0311 |
| CKMB | 0.0068 | 1.6447 |
| Killip classification | 1.1180 | 1.2382 |
| Having Spouse | -1.3067 | 1.0022 |
| (Intercept) Coefficients: -11.5174 | | |
| Degrees of Freedom: 12758 Total (i.e. Null);  12751 Residual (1 observation deleted due to missingness) | | |
| Null Deviance: 1684 | | |
| Residual Deviance: 703 | | |
| AIC : 719; BIC : 779 | | |

**Supplementary Figure 1. Predicted probability of in-hospital mortality according to total nomogram score.**

The curve shows the model-predicted probability of in-hospital death as the total risk score increases, illustrating progressive risk stratification across the full score range.


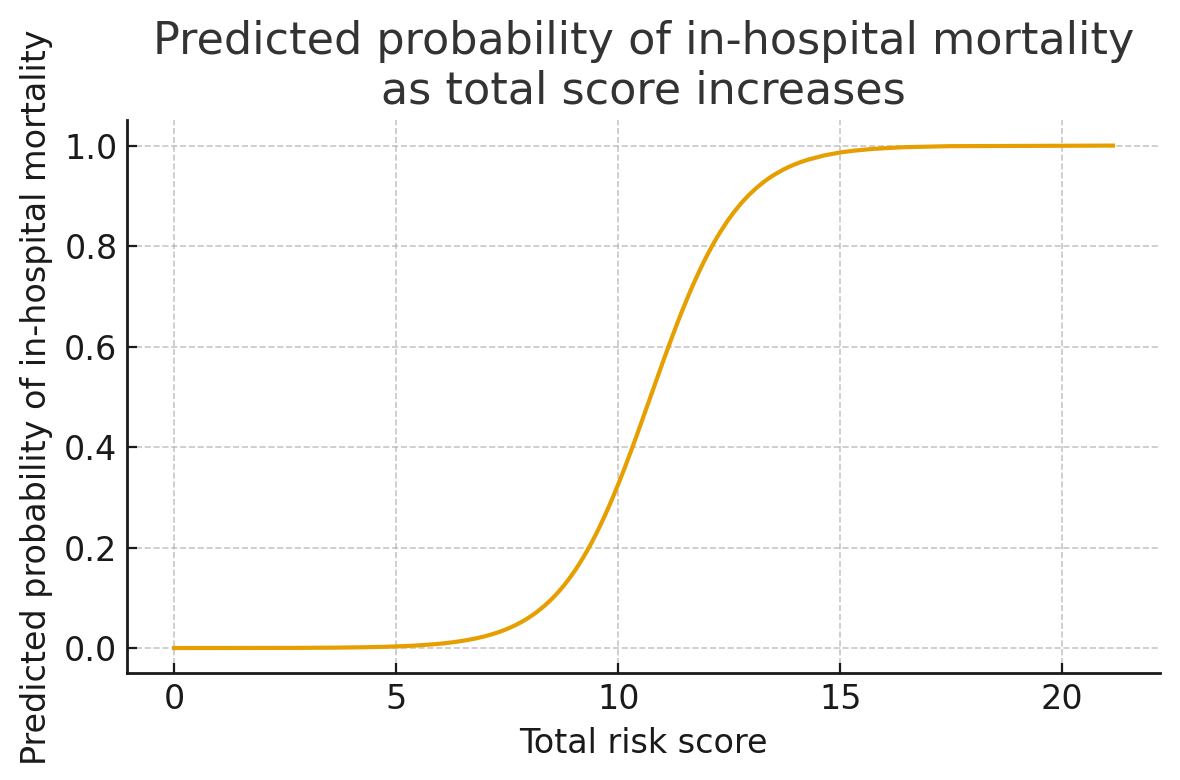

Supplement: Supplementary Table S1 — Baseline Demographic and Clinical Characteristics of ACS Patients According to Marital Status (With vs Without a Spouse). [file Datasheet1.docx]
